# Vested Interests in Addiction Research and Policy Alcohol policies out of context: drinks industry supplanting government role in alcohol policies in sub-Saharan Africa

**DOI:** 10.1111/j.1360-0443.2009.02695.x

**Published:** 2010-01

**Authors:** Øystein Bakke, Dag Endal

**Affiliations:** FORUTOslo, Norway

**Keywords:** Africa, alcohol industry, alcohol policy, developing countries, International Center for Alcohol Policies, public health, WHO

## Abstract

**Background:**

In this paper, we describe an analysis of alcohol policy initiatives sponsored by alcohol producer SABMiller and the International Center on Alcohol Policies, an alcohol industry-funded organization. In a number of sub-Saharan countries these bodies have promoted a ‘partnership’ role with governments to design national alcohol policies.

**Methodology:**

A comparison was conducted of four draft National Alcohol Policy documents from Lesotho, Malawi, Uganda and Botswana using case study methods.

**Findings:**

The comparison indicated that the four drafts are almost identical in wording and structure and that they are likely to originate from the same source.

**Conclusions:**

The processes and the draft policy documents reviewed provide insights into the methods, as well as the strategic and political objectives of the multi-national drinks industry. This initiative reflects the industry's preferred version of a national alcohol policy. The industry policy vision ignores, or chooses selectively from, the international evidence base on alcohol prevention developed by independent alcohol researchers and disregards or minimizes a public health approach to alcohol problems. The policies reviewed maintain a narrow focus on the economic benefits from the trade in alcohol. In terms of alcohol problems (and their remediation) the documents focus upon individual drinkers, ignoring effective environmental interventions. The proposed policies serve the industry's interests at the expense of public health by attempting to enshrine ‘active participation of all levels of the beverage alcohol industry as a key partner in the policy formulation and implementation process’.

## INTRODUCTION

Increasing alcohol consumption and public health problems related to alcohol in African states point to the need for the development of effective national alcohol policies. In a number of sub-Saharan countries the drinks industry has begun to assume a significant role in designing national alcohol policies. Such activities have already resulted in industry-orientated draft policies in several countries and, reportedly, the adoption of a national policy in at least one country, Lesotho. We came across documents from Lesotho [1], Malawi [2], Uganda [3] and Botswana [4] whose similarity suggested that the alcohol industry might have had significant influence on the alcohol policy development process of various low-income countries. Accordingly, we sought to investigate the process by which the documents were produced, including a review of news reports and other sources from the region [5–8]. In this paper, we describe the results of an analysis of alcohol policy initiatives sponsored by SABMiller and the International Center for Alcohol Policies (ICAP), using case study methods. The intention is not to denigrate the involved governments' sincere objective to formulate much-needed policies, but to discuss the role of vested interest in the development of such policies.

## METHODS

### Acquisition of documents

The four draft national alcohol policy documents reviewed were obtained through non-governmental organization (NGO) participants in the consultation process described later. The Malawi document was the first brought to our attention and indications of its industry origin spurred our interest. Later, the apparent similarities of the documents called for further investigation. Searches in internet-based news media from the region helped to complete the picture. Further contacts were made with NGO representatives in person and through e-mail contact. Several presentations from an ICAP Africa Regional Conference in Dar es Salaam, Tanzania, in early September 2008 indicated that SABMiller and ICAP were involved in these policy processes. Presentation at a conference organized by the Norwegian-based international development organization, FORUT (Campaign for Development and Solidarity), in Malawi in November brought out more information. Comments were sought on an earlier version of this manuscript from the two facilitators whose roles in the process are discussed later.

### Analysis

We conducted a comparative analysis of the content of the industry policy proposal. The two documents from Lesotho and Malawi [1,2] were similar to the extent that a meaningful electronic comparison was possible. In the Uganda document [3] the structure was different, and the Botswana document [4] was in a different file format, which made a manual comparison more convenient. In the electronic comparison irrelevant changes were left out (table of contents, numbering and formatting). Substitution of one wording with another was counted as one change, the same for single insertions or deletions and for moved paragraphs. The manual comparison was a qualitative analysis in order to give an indication as to the similarity between all four documents.

We also reviewed the documents against the yardstick represented by the World Health Organization (WHO)-sponsored international evidence base [9,10] and against a publication often promoted by the alcohol drinks industry and their social aspect organizations [11].

The sources described and the outcome of the analysis is the starting-point for the Discussion section. While this methodological approach provides a good ground for discussing the outcome of the process (the draft policy documents), it has its weakness in describing what transpired during the policy workshops. The additional sources provide some additional information on how the workshops have been described in the news media and by some of the participants.

## FINDINGS

### Alcohol policy development process

The draft policy documents we reviewed describe their origin as having ‘been developed as a result of broad consultations including two national symposia attended by senior representatives of Government Agencies, Non-Governmental Organizations, the beverage alcohol industry and representatives of civil society’[1–4].

The properties information of two of the MSWord documents [1,2] indicated that the ‘author’ was ‘mramsay’ from the ‘company’ SABMiller Africa Asia, possibly Mitch Ramsay, Policy and Issues Manager, SABMiller Africa. His later comment that ‘at the invitation of the workshop attendees, I was invited to prepare a record of the workshop policy proposals in local policy format’ may explain this (Ramsay M., unpublished observations). The Uganda MSWord document [3] did not give information about the ‘author’, neither did the Botswana pdf document [4]. The Lesotho [1] and Malawi [2] documents are 35 and 38 pages long, respectively. After electronic comparison 108 changes were counted, an average of three per page, varying from changes in single words to whole paragraphs. Of these changes:

1. 47 (43.5%) were changes in the name of the country and country specific information.
2. 23 (21.3%) were minor changes in wording, for example: ‘burden of’ replaced by ‘harm due to’.
3. 12 (11.1%) were paragraphs or sequences of wording which were moved in the document.
4. 26 (24.1%) were changes carrying substantive meaning, from smaller changes such as removing ‘and other risk population’ from a sentence to more substantial changes; for example, removing ‘Excessive single occasion drinking produces significant’ from a sentence about harm from intoxication [1,2].

All four documents have the same core of policy measures and some key formulations that we would expect the alcohol industry wants to see included. The Lesotho [1] and Malawi [2] documents are almost identical. The Botswana document [4] is also very similar to the two discussed above. Some sections were moved, some removed and other changes had been incorporated. The Uganda document [3] differed somewhat more from the three others. Many of the paragraphs are the same, although the structure is somewhat different. A description of the Uganda process by one of the NGO participants is consistent with this view. The initiative to develop an alcohol policy came from the Ugandan parliament and the task was entrusted to the Ministry of Health. The alcohol industry came on board only later [12]. In the other cases the initiative seems to have come from the drinks industry. Almost 2 years after the Botswana document was first drafted it resurfaced when Seretse Khama Ian Khama, the new president of the country, suggested raising alcohol taxes in mid-2008 [13–15].

The documents describe that senior representatives of government agencies, NGOs and representatives of civil society groups have been invited to attend workshops and consultations [1–4]. Reportedly, the workshops were facilitated by Mitch Ramsay of SABMiller and Mr Keith Evans. In another capacity Mr Evans is the Director, Primary Health Care and Drug Strategy, South Australian Department of Health. He is also listed as a consultant to the Washington-based International Center for Alcohol Policies (ICAP). ICAP is funded by the largest multi-national beverage alcohol producers (including Anheuser-Busch InBev; Asahi Breweries; Bacardi-Martini; Beam Global Spirits & Wine; Brown-Forman Corporation; Diageo; Heineken; Molson Coors; Pernod Ricard; and SABMiller) to operate as an agent for industry interests in global and national policy arenas. Neither the draft policy documents nor the other sources analysed indicate clearly whom Mr Evans represents.

According to Mr Evans himself, he acted as an independent facilitator in the workshops at the request of the relevant government departments of the countries concerned. He reports to have made it clear at the beginning of each workshop that he did not represent the Australian or South Australian governments, nor that he represented the views, opinions or policy perspectives of the beverage alcohol industry (Evans K., unpublished observations). Nevertheless, when two of the documents acknowledge the contribution of the stakeholders, the passages include the ‘Consultant and facilitator from Government of South Australia’[1,4]. A newspaper report from a similar event in Ghana also refers to ‘Keith Evans, special consultant of the Australian government’[16]. The Uganda document refers to support from ‘International Centre for Alcohol Policies’[3], and a presentation by one of the participants in the process in Uganda states that among others the process was facilitated by ‘[ … ] a policy Expert from ICAP [ … ]’[8]. A newspaper article from Malawi points out that Mr Evans, the facilitator of the policy development, ‘is also a Senior Consultant for International Centre for Alcohol Policies’[17]. The last draft document has left the Acknowledgement section open [2]. In personal communication and later written comments (unpublished), Mr Mitch Ramsay, Policy and Issues Manager of SABMiller, informed that SABMiller made it possible for Keith Evans to facilitate the workshops. Having taken all this into account, it is legitimate to ask if he should be considered a representative of the industry. In addition SABMiller 2008 annual report states that: ‘In Africa we are working with several governments, NGOs and public health organisations to develop national alcohol policies to reduce alcohol-related harm. As a result of these efforts, Lesotho adopted its first national policy in October 2007. Policies are nearing completion in Swaziland, Uganda, Zambia, Malawi and Ghana’[18].

Questions have been raised about the breadth and inclusiveness of the consultative process that ‘developed’ (or approved) the policies. The two drafts that acknowledge the participants [1,4] in the process list 15 and 24 participants, of whom approximately 40% are industry representatives. Fewer than 20% represent NGOs/civil society. The NGO participation also appears to have had little influence on the drafting process.

### Prominent role of alcohol and the industry

The policy drafts reflect a consistent emphasis on the role of alcohol in society and the legitimacy of industry participation in the development and implementation of national alcohol policies. For example, the Lesotho draft policy begins with the premise that the National Alcohol Policy ‘recognises the role alcohol plays in Lesotho, both in terms of its social and economic contribution and in terms of its significant capacity, when misused, to impose unacceptable costs on individuals and the community as a whole’[1]. This and other documents emphasize specifically that: ‘[t]he Government acknowledges that alcohol enjoys popularity and a place of significance in Basotho society. Alcohol when used in moderation has a positive role to play in socialisation and the industry is a major contributor to the economy of Lesotho’[1–4]. One of their key guiding principles enshrines ‘the right of the alcohol industry to conduct legitimate and legal business in a responsible way’[1,2,4].

Although the policy documents recognize the negative impact of alcohol use, the availability of alcohol seems to be one dominant theme: ‘the need to protect the reasonable expectations of adult citizens of Lesotho to purchase and consume alcohol in a safe and well regulated manner’[1].

The last part of the draft guidelines for implementation places the responsibility for implementation of the policy with a ‘National Alcohol Council’, on which representation is reserved for the industry: ‘[The Council] will draw its membership from Government Officials, representatives of the academic and Public Health Community, representatives of the Non-Government Sector and Civil Society and representatives from the Beverage Alcohol Industry’[1–4]. According to the proposal, the council will also be responsible for monitoring and for reviewing the National Alcohol Policy every 4–5 years. The policy drafts state the reason for the industry's integral involvement in the policy process by citing its ‘vested interest’ in reducing alcohol misuse: ‘The Government will encourage active participation by all levels of the beverage alcohol industry as a key partner in the policy formulation and implementation process. The beverage alcohol industry has a vested interest in ensuring that alcohol misuse is substantially reduced, and has a unique capacity to access those responsible for promoting and selling alcohol as well as to those who consume their products’[1–4].

### Suggested policy measures

According to the facilitators a tripartite model addressing supply, demand and harm reduction strategies was presented (Evans K., unpublished observations) and there were ‘significant discussions about population-based measures and references to relevant WHO sponsored research’ (Ramsay M., unpublished observations). Whatever the content of these discussions in the workshops, the draft national alcohol policies take essentially the same approach proposed in ICAP's publication *Drinking in Context*, where the emphasis rests upon the need to manage drinking patterns and strengthen industry/government/public health partnerships [11]. The industry's use of key words such as ‘culture’, ‘context’, ‘patterns’ and ‘partnerships’ to describe the need for narrowly targeted interventions, rather than population-based prevention measures, draws attention away from two of the most effective policy responses—control of availability and taxation [9]. On the other hand, the WHO-sponsored research compendiums *Alcohol in Developing Societies: a Public Health Approach*[10] and *Alcohol: No Ordinary Commodity*[9] summarize present knowledge of cost-effective interventions to reduce alcohol-related harm, and recommend a set of best practices [19]. [The best practices recommended by Babor *et al*. are minimum legal purchase age, government monopoly of retail sales, restrictions on hours or days of sale, outlet density restrictions, alcohol taxes, sobriety check-points, lowered blood alcohol concentration (BAC) limits, administrative licence suspension, graduated licensing for novice drivers and brief interventions for hazardous drinkers.] The draft policy documents are devoid of any reference to *Alcohol: No Ordinary Commodity* or other compilations of the international evidence base on alcohol prevention developed by independent alcohol researchers working on behalf of the WHO [20]. Only a few of the best practice recommendations are included in the set of six policy priority areas given in the documents (five policy areas in the Uganda document). Prominence is given rather to measures that have not been proved effective in changing alcohol consumption and harm. Some examples are given below.

Under priority area one—intoxication—the draft policy's first option is to increase public awareness and understanding through educational programmes, and messages focusing upon personal responsibility and moderation. Although the documents prescribe briefly ‘review of’ and ‘compliance with’ current legislation dealing with alcohol or the impact of the misuse of alcohol, the documents lack specific reference to any existing legislation in the alcohol field in three of the countries or any discussion of how these could be implemented more effectively. One exemption is the Uganda document which points to the need to review the Enguli Act [3].

Priority area two—public safety and amenity—includes an objective to develop a national plan to address drink driving, but recommends only two measures to reduce accidents from the combination of alcohol and driving: ‘Review Blood Alcohol Content (BAC) limits for all drivers; and Provide alcohol-related brief interventions, treatment and rehabilitation support for drink drive offenders’[1]. Although the draft proposals note the need to review BAC limits, it is significant that the documents fail to recommend proven policies to require random breath testing of drivers on the road and swift punishment of those convicted of drinking-driving offences.

Priority area three—health impacts—starts by pointing out the ‘positive and negative health impacts from alcohol consumption’[1–4]. The relevance of positive health benefits from drinking may be questioned, considering that the possible association of moderate alcohol consumption with health benefits (in studies coming from the developed world) has been found primarily in older people. The likelihood that such benefits would be available in a developing country such as Malawi, where life expectancy is currently below 40 years, is slim. In addition, recent reviews have also shown that due to systematic bias in observational studies there may be no health benefits in any age group [21].

Priority area four—patterns and availability—basically upholds the need to ‘develop and implement a transparent self-regulatory system by the alcohol beverage industry’ and conduct public education campaigns [1–4].

In this section there is also a mention of the need to regulate alcohol promotions concluding by subscribing to the preferred industry approach: ‘The Government supports the need for self-regulation by the alcohol beverage industry as the most suitable way to manage marketing and promotions’[1,2,4].

Priority area five—at-risk populations—illustrates the document's strong focus upon the individual: ‘Individuals who are at increased risk for harm from drinking require special attention with regard to prevention and intervention measures’[1,2,4].

In priority area six—research—the need for more evidence is mentioned.

In a similar National Alcohol Policy developed by the government of Kenya [22], despite industry involvement, the resulting national alcohol policy reflects more strongly the international evidence base on alcohol prevention developed by independent alcohol researchers working on behalf of the WHO.

### Low-income countries context

In a developing country context it is necessary to look at the relationship of alcohol and poverty. A study from Sri Lanka illustrates the nexus between alcohol use and severe social, economic and health effects for people living in poverty. It concluded, in part, that ‘Alcohol, and the consequences of alcohol use, influence greatly the every day life of poor people. Not only are the lives of those who drink severely affected, but perhaps even more, the lives of others such as their wives and children’[23]. A recent Policy Brief from the Chronic Poverty Research Centre in Uganda, ‘Drinking into deeper poverty’, paints a similar picture: ‘Excessive alcohol consumption is one of the key drivers and maintainers of poverty especially in the rural countryside’[24].

Although, according to Mr Ramsay (unpublished observations), social and economic deprivation were reportedly discussed in the workshops, those perspectives are not addressed in the policy documents, nor are other specific challenges to developing countries such as alcohol and its relations to key issues such as human immunodeficiency virus/acquired immune deficiency syndrome (HIV/AIDS), gender-based violence, child rights and others [25–28].

Scarcity of qualified health workers is a challenge in many of these countries. For example, in 2004 Malawi had 266 physicians in a population of close to 13 million people [29]. However, one of the two policy objectives for priority area three—health impact—is to ‘Develop and implement a national plan for ensuring that evidence-based assessment and treatment services for alcohol misuse and dependence are available in urban and rural Malawi’[1], with similar wording in the other three documents [2–4].

## DISCUSSION

A review of the various policy proposals from several sub-Saharan countries leads to the conclusion that, rather than originating from discussions among participants at national workshops, the documents share the same source—designed, initiated and organized by industry interests. They are all virtually the same in wording, structure, even page formatting. A few paragraphs appear in different places and obviously the names of the countries have been changed. Fewer than 25% of the changes made between the two documents from Lesotho and Malawi are substantive changes with any significance for the content. Nevertheless, these changes do not reflect any adaptation to the local context or indications of a participatory process. The two other documents are also very similar.

The enormous market power exercised by the alcohol companies can translate easily into political power [30]. The policy development processes and the proposed policy drafts provide insights into the working methods of some of the multi-national drinks companies. One point of particular concern is the acknowledgements in two of the draft documents to the ‘Consultant and facilitator from Government of South Australia’[2,4], when we have been informed that Mr Evans' participation was made possible by SABMiller (Ramsay M., Bakke Ø., personal communication; and unpublished comment). These issues may lead easily to questions regarding his role as an independent consultant.

In 2006 the WHO convened an expert committee, which submitted policy recommendations in April 2007 [20]. Those recommendations are almost entirely absent in the policy drafts proposed throughout Africa. In fact, at one point the drafts seem to be a direct rebuttal to the experts' recommendations for the industry's role in the development and implementation of alcohol policy. The Committee Recommendation 9 calls upon WHO to ‘continue its practice of no collaboration with the various sectors of the alcohol industry. Any interaction should be confined to discussion of the contribution the alcohol industry can make to the reduction of alcohol-related harm only in the context of their roles as producers, distributors and marketers of alcohol, and not in terms of alcohol policy development or health promotion’[20].

Few, if any, would accept Philip Morris as the designer of the tobacco policy for a national government. The alcohol industry's current policy proposals in several Southern African states can hardly be viewed any differently.

We have documented above how the focus of the industry's draft policy is towards the economic and social contribution of alcohol in the society. The key issues and policy measures in the documents indicate that they promote industry self-regulation, for instance with regard to marketing. Their fear of restrictions on alcohol advertising and other marketing activities at a time when the multi-national beer producers are increasing their presence in many African countries might be an important impetus for the present industry initiatives. As long as they can relegate marketing activities to self-regulation, they can use advanced marketing techniques to promote drinking among new segments of the population in these countries.

The policy proposals take an individualistic approach rather than considering public health strategies. ‘Soft’ unproven prevention measures involving consumer education and information dissemination are promoted, rather than control policies, which have the strongest evidence base as analysed by Babor *et al*. [9,10]. With this bias the draft National Alcohol Policy documents will be ineffective in reducing harm from alcohol. They are more likely to fuel the industrial epidemic of alcohol harm [31] that has already made alcohol the number five risk to health, according to the Global Burden of Disease study [32].

Despite their alignment with the approach proposed in ICAP's publication, *Drinking in Context*[11], the draft National Alcohol Policy documents fail to meet ICAP's own standards that ‘recognize socio-cultural differences’. They contain only a few weak references to specific national situations and alcohol-related trends in each country and fail to address existing policies and laws as well as traditional culture. Data from the WHO *World Health Survey 2003* cited in the Malawi country profile of the WHO *Global Status Report on Alcohol* 2004 show that most Malawians either do not drink, drink very little or drink infrequently [33]. In the survey, 76.7% were recorded as non-drinkers; 58.3% among men and 90.8% among women. This is not to say that there is not an alcohol problem to be addressed in the country, but in this context it seems somewhat strange to adopt a policy that ‘will establish the basis for the place of alcohol in the lives of Malawians. It needs to move all Malawians to safer drinking patterns in shaping the future’[2].

In particular, the drafts seem to miss the special context of a developing country, and ignore well-documented local concerns and the many organizational, logistical and resource challenges involved in implementing certain policy approaches. Certain sections of the draft policy concern expanding and enhancing the capacity of the health care and related professions to address alcohol-related health problems. That approach would represent a great challenge for developed countries, let alone the already strained African health-care systems. Malawi is a case in point. It has one of the highest rates of HIV/AIDS prevalence and morbidity in the world and one of the lowest ratios of qualified health workers per capita (see above). It is hard to see how adding the growing alcohol problem to an already fragile health-care sector will be an effective and cost-effective policy response.

The timing of the Alcohol Policy initiatives in Africa is of interest. In 2005 the 58th World Health Assembly (WHA) passed resolution WHA58.26: ‘Public health problems caused by harmful use of alcohol’[34], asking the WHO to report on evidence-based strategies and interventions to reduce alcohol-related harm. In May 2008 the member states of the WHO again passed an alcohol resolution at the World Health Assembly (WHA61.4) [35]. That resolution, drafted by the group of African countries, called for a WHO-sponsored Global Strategy to Reduce Harmful Use of Alcohol. In passing the resolution, the World Health Assembly made one final clarifying change to its language, substituting a controversial phrase that suggested WHO *collaboration* with ‘economic interests’. That change restored the original wording of the African proposal, for WHO ‘t*o collaborate and consult with Member States, as well as* consult *with intergovernmental organizations, health professionals, nongovernmental organizations and economic operators on ways they could contribute to reducing harmful use of alcohol*’[35][author's emphasis].

The recommendations of the Expert Committee (above) and the decision of the World Health Assembly describe a role for the industry that may also be considered as guidelines for individual member states. These guidelines are very different from those related to the industry's vested interest discussed above and the seemingly collaborative and governing role played by some alcoholic-beverage companies in the policy processes analysed.

The timing of the policy initiative in Africa points to the likelihood that this was spurred by the new alcohol initiative at WHO in 2005. This may represent an attempt to establish policies on that continent before any WHO recommendations have a chance to influence the content of those policies. Now that the WHO global strategy development process has begun, it is conceivable that many of the involved governments will put industry-initiated alcohol policies on the shelf and await the recommendations of the WHO Global Strategy. That prudent action would serve the public health and safety interests of those nations.

We do not dispute the documents' assertion that ‘[t]he need for a sensible and sustainable alcohol policy is well understood’. None the less, the alcoholic-beverage companies' conduct, which we document in this paper, suggests strongly that the policy proposals err significantly when they proclaim that ‘The time for conflict over the best way forward is gone’[1–3].
